# Individual Features in the Typology of the Nervous System and the Brain Activity Dynamics of Freestyle Wrestlers Exposed to a Strong Physical Activity (a Pilot Study)

**DOI:** 10.3390/bs10040079

**Published:** 2020-04-20

**Authors:** Irina Polikanova, Sergey Leonov, Aleksey Isaev, Liudmila Liutsko

**Affiliations:** 1 Faculty of Psychology, Lomonosov Moscow State University, Moscow 125009, Russia; svleonov@gmail.com (S.L.); isaev_aleks@mail.ru (A.I.); liudmila_liutsko@yahoo.es (L.L.); 2 Radiation Department, ISGlobal, 08003 Barcelona, Spain

**Keywords:** fatigue, physical exertion, freestyle wrestling, EEG, spectral characteristics, Leonhard–Shmishek questionnaire, Spielberger–Hanin questionnaire

## Abstract

Nowadays, knowledge of psychophysiological features, particularly on the nervous system’s characteristics, is essential in the sporting context, particularly for freestyle wrestling. The study aimed to investigate the peculiarities of the wrestlers’ nervous system—on the individual and electrophysiological levels in two functional states—in calm wakefulness and during intense physical fatigue. Psychological (Well-being, Activity, Mood; Spielberger–Hanin; Leonhard’s questionnaires), as well as electrophysiological techniques (dynamics of the dominant and average frequencies of the main electroencephalogram (EEG) spectra—theta, alpha, low and high-frequency beta rhythms), were used in the study. It was shown that athletes were mainly characterized by the hyperthymic type of character accentuation and a low frequency of theta rhythm in a calm wakefulness state. After the acute physical load, wrestlers with high hyperthymia showed a moderate increase in theta, whereas other athletes showed a decrease in this parameter. Regardless of the level of hyperthymic accentuation, all wrestlers were characterized by an increase in the frequency of alpha rhythm after exercises in the left hemisphere. These results suggest the existence of a particular functional system in freestyle wrestlers, which allows the body’s regulatory systems to be adapted for the effective implementation of sports activity.

## 1. Introduction

The physical nature of fatigue is complex. Numerous facts indicate that the main process leading to the occurrence of fatigue is the gradual suppression of the activity of the central nervous system and the development of inhibition. Fatigue leads to depletion of the internal resources of the body, and this forces it to switch to less effective ways of functioning, which affects the work of various body systems. This can be manifested in an increase in heart rate, as well as in a decrease in the strength and speed of muscle contractions, dis-coordination in the work of regulatory systems, difficulties in developing and inhibiting conditioned reflexes. As a result, the pace of work slows down, accuracy, rhythm, and coordination of movements are disturbed [1,2].

Speaking of physical exhaustion, it is important to note that it can manifest itself at the central and peripheral levels. The latter, as a rule, is manifested in behavioral reactions. At the same time, central fatigue is associated with a decrease in activation in the primary motor areas of the cerebral cortex associated with the innervation of skeletal muscles, and accordingly with a gradual decrease in the physical ability of its activation [3,4].

Studies of electrophysiological indicators of physical fatigue are divided as a rule into two main types—comparing the electroencephalogram (EEG) recording done directly during exercise with the EEG recording in the background state (quiet wakefulness); and in cases when EEG recording directly during exercise is difficult, a comparison of the dynamics of electrophysiological indicators before (background state) and after exercise is made.

Free-style wrestling, in particular, is a kind of sport where high-quality EEG recording is difficult directly during a specific sports activity. This is the kind of sport consisting in conducting a duel between two athletes according to certain rules and using various methods of wrestling (captures, throws, sweeps, etc.) to put an opponent on the shoulder blades or to perform a greater number of effective techniques/holds and, thus, to win technically.

Since freestyle wrestling is a complex coordination sport requiring from athletes not only the development of physical qualities (strength, agility, speed, flexibility, endurance) but also psychophysiological ones (the use of cognitive resources to make decisions about choosing actions, speed of reaction, features of attention processes under the conditions of time deficiency, etc.), it can be assumed that the peculiarities of the wrestlers’ nervous system may influence the efficiency of an athletes performance. In particular, the mobility of the nervous system in freestyle wrestling is the most important and fundamental element of an athlete’s ability to predict [5]. Even imbalance often does not prevent an athlete from attaining mastery if he has a mobile nervous system. Wrestlers with an inert nervous system have more difficulty in showing the ability to improvise during the tactical struggle; they are slow to adapt when the opponent changes the pattern of tactical actions.

The presence of high lability and mobility of nervous processes determine the speed of perception and processing of information, the effectiveness of integrative brain activity, and the preparation of athlete responses. Without all these, it is impossible to carry out effective training and competitive activities in such sports, such as judo or freestyle wrestling, because, in addition to assessing the current situation, athletes need to develop the ability to foresee its possible changes, i.e., anticipation [6,7,8,9,10,11].

In the study of the properties of the nervous system in students practicing judo, Shakhanova and colleagues [12] showed that the properties of lability, mobility, and speed of the nervous system might change during lifetime under the influence of certain factors; in particular, the authors showed that as athletes mature and increase fitness, their simple visual-motor reaction is shortened which characterizes the mobility of the nervous system, whereas this tendency was not found in the students who were not involved in sports.

A similar study was conducted by Berdichevskaya and colleagues [13] on young boxers. In the framework of this work, the typological properties of the nervous system of boxers and untrained youths were compared. The authors showed significantly lower reaction time in young boxers in comparison to non-athletes, which indicates a greater mobility of the nervous processes in them. In addition, the authors investigated the level of functionality parameter, which characterizes the strength of the nervous processes. Young boxers were characterized by high values of this parameter. No differences were found among the athletes and non-athletes in terms of equilibrium of the nervous processes, determined by the indicator of the stability of the reaction. The differences in the typological properties of the nervous system of young male boxers identified by the authors can largely determine the success of their activities. The greater the strength of the nervous processes, the stronger the volitional and motor manifestations are; mobility of nerve functions provides quick processing of imagery-motor information and the ability to move from one state to another [14,15]. The study of Berdichevskaya with colleagues also showed that young boxers, unlike non-athletes, are characterized by higher indicators of short-term and figurative memory [13]. The authors explain this by the specifics of the sports activities of boxers, who develop tactical thinking along with the improvement in motor skills. In this regard, the volume of attention distribution is important in all types of martial arts, which is associated with the need to respond to any sudden signals [16,17] quickly.

Emotionality and intensity are the indispensable conditions for the flow of tactical thinking in wrestlers during competitive activities. The athlete has to make decisions in the context of mental tension, acute situations of struggle with a rival, facing the constant threat of erroneous action to nullify the efforts of many training sessions [18].

The activity of an athlete in wrestling can be considered as the activity of an operator in difficult-to-manage systems since freestyle wrestling is a large combinatorial situational system with a high degree of uncertainty of events [19]. An operator involved in complex control systems often has to comprehend the problem situation, identify a specific task, and find solutions during a limited time. Being late in these conditions is equivalent to an error and can lead to complications of the problem situation and sometimes to partial or complete disruption of the system. Thus, the identification of electrophysiological EEG indicators, reflecting the most optimal functional states for each particular sport, and for wrestling, in particular, will allow the effectiveness of training athletes to increase.

Psychophysiological studies of fatigue that occur after physical exercise show a correlation with a number of EEG parameters, as well as a significant difference in the dynamics of such parameters among professional athletes and beginners. Popova [20], in the experiments with kick-boxers on Mosso ergograph, showed that professional athletes are characterized by a significantly greater pronounced alpha rhythm compared with the control group (non-athletes). The authors associate these results with less development of fatigue in athletes.

Taking into account all of the above, we can see that free-style wrestling requires from athletes not only the development of physical qualities but also psychophysiological ones, such as features of the nervous system. The presence of high lability and mobility of nervous processes determine the speed of perception and processing of information, the effectiveness of integrative brain activity, preparation of athlete responses. These features of the nervous system are reflected both at the behavioral level and at the electrophysiological level (dynamics of EEG activity). Information about the characteristics of the nervous system of free-style wrestlers will help to better adapt the training process depending on the individual characteristics of the athletes.

For this purpose, we carried out a pilot study aimed to investigate the peculiarities of the wrestlers’ nervous system, on the subjective and electrophysiological levels. To better show the features of the dynamics of nervous processes, we investigated two functional states—in calm wakefulness and during strong physical fatigue.

The aim of our work was to study the dynamics of the functional state of freestyle wrestlers before and after increased physical activity using psychological (Well-being, Activity, Mood (WAM); Spielberger–Hanin questionnaires, and Leonhard’s method (Shmishek’s modification) of studying personality accentuation) and electrophysiological techniques. 

## 2. Materials and Methods

### 2.1. Participants and Methodology 

Nine athletes of the Moscow State University freestyle wrestling team took part in our study—with degrees of Candidates of the Master of Sports and the Masters of Sports (average age—22 years; range: 19–32, 100% male). All of them participated on a voluntary basis with the previously signed consent, already approved by the Ethical Committee of the Russian Psychological Society (112/02). The exercise took place in the training environment of preparing athletes for competitive activities. Before training, the athletes filled out test forms (WAM, Spielberger-Hanin, and Shmishek–Leonhard methods), after which background EEG recording was measured.

By results of Leonhard’s method (modification of Shmishek) of studying the accentuation of the personality, our participants were divided into two groups according to the results of the “Hyperthymia” scales: Group 1 include six athletes with a high level of hyperthymia accentuation and group 2 include three athletes with normal hyperthymia. 

The electroencephalographic examination consisted of EEG background recording with open eyes (BOE)—2 min and EEG background recording with closed eyes (BCE)—2 min and was performed before and after increased physical activity. Thus, our study design was: 2 sensory conditions (BOE -open/BCE -closed eyes) × 2 levels of physical activity (no physical activity (calm wakefulness)/increased physical activity) × 2 groups (with a high and moderate level of hyperthymia).

The physical load consisted of the following exercises: athletes conducted warm-up starting with running, running exercises, general developmental exercises, strength exercises, special wrestling exercises, and stretching exercises. Its duration was approximately 25–30 min. Further, the moves were practiced in the stand (15 min), wrestling in different positions (10 min), *par terre* wrestling (6 min), the practice of throws (8 min), and wrestling in the stand for all moves (5 min). After the main exercise on the carpet, the athletes underwent tests for strength endurance, which consisted of the following exercises: push-ups, an exercise for the abdominal press (book), jumping up, and “throwing off legs”. Each exercise was performed for 15 s alternately (3 laps—3 min), 30 s break, and then the same exercises for three more laps (total 6 min of work).

### 2.2. Psychological Questionnaires

Well-being, Activity, Mood (WAM) is a 30-items test [21]. This test is composed of three subscales (10 items for each state) to assess subjective functional states (Well-being, Activity, and Mood), rated by a 7-point Likert scale with negative and positive poles. The mean value is calculated for each subscale. The average score on the scale is 4, values above this signify the optimal psychological state, values below that indicate the opposite.

Spielberger–Hanin inventory is a 40-item test [22] aimed to assess the state anxiety level (reactive anxiety, as a condition) and the trait anxiety (as a stable personal characteristic). Designed by Ch. D. Spielberger and adapted to Russian by Hanin [23]. This test consists of two subscales—state anxiety and trait anxiety (20 items for each subscale), all rated on a 4-point Likert-type scale (1 = “not at all” to 4 = “very much likely”). Total score is calculated for each subscale and varies from 20 to 80 points. Moreover, the higher the final score, the higher the level of anxiety (state or trait). Scores under 30 points indicate the low level of anxiety; scores ranged 31–44 indicate moderate anxiety, and scores above 45 points indicate high anxiety.

The Leonhard questionnaire (adapted by Shmishek) is a personality questionnaire aimed to identify personality accentuations [24]. This inventory consists of 88 items (that should be answered in “yes”/“no” mode) and composed of 10 subscales representing each of personalities accentuations: demonstrative, pedantic, stuck, excitable, hyperthymic, dysthymic, anxious-fearful, cyclothymic, affective, emotive. The maximum score for each accentuation (on each scale) is 24. Accentuation is considered preset if a score on a scale is 12 and above.

### 2.3. EEG Recording

EEG recording was performed using a 21-channel electroencephalograph performed by Medicom MTD in accordance with the “10–20” international system, which establishes the exact location of the electrodes on the scalp by reference ear electrodes. The quantization frequency was 500 Hz. Electroencephalogram (EEG) was recorded from 16 standard electrodes of the frontal (F), central (C), temporal (T), parietal (P), and occipital (O) regions of both hemispheres of the neocortex relative to the ear reference electrodes.

### 2.4. Data Analysis

Statistica 8 (for Windows, V 8.0, StatSoft) was used for statistical data analysis. A *t*-test was used for dependent samples. In the case of comparing the groups with high and low hyperthymia, a nonparametric Mann–Whitney criterion was used due to a small sample. 

Statistical data analysis was performed for the following *indicators* (EEG rhythms ranges presented in Table 1):

- the dynamics of the dominant frequencies of theta, alpha, low frequency (LF) beta, high frequency (HF) beta rhythms—a comparison of background readings before and after exercise;
- dynamics of average frequency of alpha, low frequency (LF) beta, high frequency (HF) beta rhythms—comparison of background values before and after exercise.

For the analysis of EEG, the segments of background EEG 20 s in duration and with the lowest content of artifacts were used.

The dominance of each EEG rhythm is not evenly distributed over the scalp due to the involvement of different subcortical structures in the generation of the corresponding rhythm. Therefore, EEG analysis was carried out for each selected rhythm in certain electrodes for the left and right hemispheres, respectively, in accordance with the literature on the subject. Thus, the analysis of the theta rhythm was conducted for the electrodes F3, Fz, and F4, for the alpha rhythm analysis, in electrodes P3, Pz, and P4, and for beta rhythms, in electrodes C3, Cz, and C4.

For the WAM psychological questionnaire, mean values and standard deviations were calculated.

## 3. Results

### 3.1. Subjective Indicators

#### 3.1.1. WAM Method

Statistical analysis showed a significant decrease in scores on all scales of the WAM questionnaire, which may indicate the development of fatigue processes at a personal level: “general feeling of health” (5.8 vs. 4.2, *p* ≤ 0.01), “activity” (5.4 vs. 4.3, *p* ≤ 0.01), “mood” (5.7 vs. 5.1, *p* ≤ 0.05).

#### 3.1.2. Spielberger–Hanin Questionnaire

This technique did not reveal significant changes in the indices of situational anxiety among freestyle wrestlers after increased physical activity.

#### 3.1.3. Shmishek–Leonhard Questionnaire

This questionnaire revealed the predominance of accentuation “Hyperthymia” in our sample (in 6 out of 9 wrestlers) (Appendix A).

According to these results, we divided our sample for group 1 (with high hyperthymia; 6 subjects) and group 2 (normal and low hyperthymia; 3 subjects). We compared both groups according to the studied indicators. 

In particular, group 2 is characterized by a significantly stronger decrease in the WAM scale “activity” after physical exertion compared to group 1 (Table 2).

The comparison of group 1 and group 2 by the Spielberger–Hanin questionnaire did not reveal any significant differences. 

### 3.2. The Results of Electrophysiological Studies

#### 3.2.1. Theta Rhythm

Analysis of the dynamics of theta rhythm in wrestlers before and after intense physical activity showed a statistically significant decrease mainly in the right hemisphere (electrode F4) in the eyes-closed state (BCE) both for dominant (5.57 vs. 4.14, *p* ≤ 0.05) and average frequency (5.63 vs. 5.19, *p* ≤ 0.05).

In addition, the same trend has been shown for the central electrode (Fz), but only for the dominant frequency of theta rhythm (5.29 vs. 4.46, *p* ≤ 0.05) and left hemisphere (F3) for the average frequency (5.56 vs. 5.18, *p* ≤ 0.05) (Table 3).

The comparison of group 1 (high hyperthymia) and group 2 (low hyperthymia) by parameters of theta rhythm showed the trend to decrease for both groups after intense physical activity. But group 1 is characterized by initially (in calm wakefulness state) a lower dominant frequency of theta rhythm predominantly in the left hemisphere (electrode F3) compared to group 2. In addition, group 1 is characterized by a tendency to increase theta in the eyes-open state, whereas group 2, by the decline of this parameter (Table 4).

#### 3.2.2. Alpha Rhythm

Analysis of alpha rhythm dynamics in wrestlers before and after intense physical exertion showed a statistically significant increase in the eyes-closed state, mainly in the left hemisphere (electrode P3) (Table 5).

In addition, for an eyes-open state, there is a significant increase in the average frequency (electrode Pz) (Table 5).

The comparison of group 1 (high hyperthymia) and group 2 (low hyperthymia) by parameters of alpha rhythm did not reveal any significant differences. 

Analysis of the remaining EEG frequency ranges did not reveal significant changes in their dynamics after intense physical exercise. 

## 4. Discussion

Our study showed that freestyle wrestlers were mainly described by a hyperthymic type of character accentuation, characterized by high activity and vigor [25].

The results of the study also showed that among six participants with pronounced hyperthymia, there were four athletes who had a pronounced accentuation of affective–exalted type (Appendix A). This type is characterized by a wide range of emotional states. Exaltation (enthusiastically excited state) manifests itself in an explosive reaction to stimuli, alternation of directions of activity, high impressionability, strong attachment to friends [26]. At the same time, high indices on hyperthymia and exaltation scales may indicate an unusually radiant (even extravagant) behavior, enthusiasm, and demonstrativeness.

It is important to note that despite the fact that the experiment was conducted as part of the training process, the physical load placed on the athletes was of high intensity. Besides, the EEG recording was carried out in close proximity to the training hall, which provided the minimum time delay between the end of the training session and the EEG recording.

Statistical analysis of the dynamics of electrophysiological parameters showed a significant decrease in the frequency of theta rhythm (dominant and average) predominantly in the right hemisphere after intense physical exertion, as well as a substantial increase in the frequency of alpha rhythm in the left hemisphere (dominant and medium).

The obtained results are generally consistent with literary data. Japanese scientists [27] conducted an magnetoencephalography (MEG) study that showed a decrease in the power of the alpha rhythm during physical fatigue (mainly in the ipsilateral sensorimotor region and the prefrontal cortex), and its increase after physical fatigue caused by maximum repeating squeezing of the expander in the left hand during 10 min. The authors associate this phenomenon with the participation of the alpha rhythm in information processes, including the anticipation processes, which is manifested in the de-synchronization of the power of the alpha rhythm [28,29]. In the authors’ opinion, the increase in alpha rhythm is associated with a weakening of anticipation processes and appropriate synchronization.

Studying physical fatigue using a bicycle ergometer (not with professional athletes) Taiwanese scientists [30] showed that as fatigue develops, an increase in the average power in each frequency band of the EEG spectrum (alpha, beta, theta) is observed. At the same time, the subjects with a moderate degree of fatigue (separation by the degree of fatigue was carried out according to ECG indexes), showed much faster recovery after the cycle ergometer, whereas for the group with strong fatigue, the restoration of the power of the main EEG rhythms took more time.

Of particular importance may be the results obtained by us in the study of groups with high and average levels of hyperthymia (six people and three people, respectively). The group with low hyperthymia was characterized by a significantly stronger decrease in the WAM scales “health” and “activity” after physical exertion compared to a group with high hyperthymia. This may indicate that people with hypertension are less prone to fatigue at a subjective level.

In addition, it is important to note the positive relationship between the hyperthymic and exalted types of character with the mobility (lability) of nervous processes, as shown in the literature on the subject, as well as their balance [31], which is extremely important for freestyle wrestlers.

At the electrophysiological level, the group with high hyperthymia differed from the group with a normal level of hyperthymia by in calm wakefulness state initially lower frequency of theta rhythm (dominant and average). In addition, the group with high hyperthymia was characterized by a tendency to increase theta in the eyes-open state after physical load, whereas the group with a normal level of hyperthymia, by the decline of this parameter. Theta rhythm is divided into hippocampal and frontal. The frontal theta rhythm line represents spontaneous or rhythmic activity-related short flashes of rhythmic activity associated with the task in the range of 5.5 to 8.5 Hz in the frontal electrode region [32]. During the state of quiet wakefulness with eyes open or closed, the medium-light theta rhythm appeared like a noticeable peak in the EEG spectra for only a small number of subjects (approximately 43% of adult subjects (18–28 years old).) This theta rhythm was synchronized with the implementation of actions in response to significant environmental events and is associated with memory recall and coding of memory traces [32].

Mitchell’s study shows that the average theta rhythm correlates with mental effort when performing cognitive tasks [33]. In addition, the Kaplan study showed that the spectral amplitude of theta activity correlated not with the success of the task, but with its complexity [34]. Thus, the increase in theta activation is observed in the conditions of performing cognitive tasks, especially related to the processes of memory and attention. An increase in theta activity can also be observed when there is no load on the memory, for example, while waiting for a pain stimulus [35]. Neurophysiological studies in animals have shown an increase in the activity of the hippocampal theta rhythm in conditions where attention is strongly focused on specific stimulation. Theta rhythm acts as a kind of selective attention mechanism that improves the processing of information about the target stimulus, including inhibiting irrelevant information [36]. In addition, the activation of the theta rhythm also depends on the individual significance of the stimulus on which the subject’s attention is focused [37]. Thus, the activation of a theta rhythm can be interpreted as highly focused attention to a subjectively significant stimulus or event [38].

In studies of Japanese scientists, healthy subjects were divided into three groups according to the intensity of frontal theta (low, medium, and high mid-lobe theta activity) in the area of the frontal electrode (electrode Fz) during their solving arithmetic problems. The first group showed the lowest level of anxiety and neuroticism, and the highest level of extraversion. The third group showed the opposite results. There were no differences between the groups regarding the task being solved. Thus, it was concluded that the intensity of the average frontal theta rhythm was mainly related to the personal characteristics of the subjects and their level of anxiety [39].

The Harvard research team studied the relationship between frontal theta activity and glucose metabolism in the human brain, which showed the greatest activation in the anterior cingulate gyrus. Simultaneous registration of the EEG of glucose metabolism (a positron emission tomography (PET) study) was performed on a group of healthy subjects [40]. The anterior cingulate gyrus is assumed to play a role in a variety of autonomic functions, such as regulation of blood pressure and heart rate. It also participates in the performance of cognitive functions, such as expecting rewards, decision making, empathy, controlling impulsiveness, and emotions. The frontal theta rhythm correlates with mental effort when performing cognitive tasks [41].

Sports activity in freestyle wrestling is characterized by a high involvement of cognitive functions. Athletes constantly need to make decisions about the choice of response actions, attacking actions, tactics, and strategies of the fight. Such intense cognitive activity is associated with an increase in activation in the anterior cingulate gyrus, which is manifested in increased theta activity. Probably, after increased physical exertion, when fatigue begins to develop, there is a decline in the activation of the theta rhythm, and this is what we see in our case.

An increase in the alpha rhythm after exercise may be due to several factors. An increase in the activity of the alpha rhythm is classically associated with the development of fatigue, in particular cognitive fatigue [42,43,44,45,46,47,48,49,50,51,52,53,54,55,56]. In addition, the alpha rhythm is associated with the flow of information processes.

The alpha rhythm is characterized by a frequency of 8–13 Hz and an amplitude of 5–100 µV and is registered in the region of the primary or secondary sensory zones of the cortex at rest. When the sensory areas of the cortex are activated, the alpha rhythm is inhibited. In a healthy person, the alpha rhythm is registered in the region of the parietal, occipital, and sensorimotor cortex (sensorimotor rhythm, or mu-rhythm). Typical manifestations of the alpha rhythm are high-amplitude waves in the parietal and occipital electrodes. Such synchronization is caused by blocking the visual inputs to the occipital regions. Occipital rhythm usually dominates in the EEG recording during the state of quiet wakefulness with eyes closed. Sometimes scientists single out the parietal alpha rhythms (high-amplitude rhythms in the alpha frequency range in the parietal areas with a maximum in Pz), which also increase in the state with closed eyes like the occipital alpha rhythm, although some subjects can show a decrease in the parietal alpha rhythm in response to closing the eyes. The frequency of the parietal rhythm is usually lower than the frequency of the occipital rhythm, measured in the same person. The total strength of the parietal rhythms increases with the increase in the difficulty of the task and is higher, while problem-solving compared with the state of calm wakefulness with open eyes.

Thus, in the framework of the study, it was shown that freestyle wrestlers in a state of calm wakefulness were characterized by a low frequency of theta rhythm, which in turn was associated with the activity of the anterior cingulate gyrus involved in the implementation of such cognitive processes as expecting awards, decision making, empathy, control of impulsivity and emotion. During wrestling, there is a significant decline of theta rhythm and an increase in alpha rhythm, which apparently allows the functional system of an athlete to adapt to the effective implementation of sports activities due to the strain of regulatory systems.

As part of this study, we obtained results reflecting the psychological and psycho-physiological characteristics of freestyle wrestlers, as well as their dynamics in conditions of physical exhaustion. Since it was a pilot study, the sample size was not huge (but we recruited participants that consisted 45% of all wrestlers in the sports center), these results are difficult to generalize to a broad sample; however, they can be considered as a start for further work in this direction.

## 5. Conclusions

Our study of physical fatigue in freestyle wrestlers showed that a strong physical exertion affects the dynamics of a number of psychological and psychophysiological parameters. An intensive physical load significantly affected the decrease in the subjective feeling of well-being, activity, and mood in the tested wrestlers. In addition, physical fatigue was reflected in a significant decrease in the dominant and average frequency of theta rhythm in the right hemisphere after intense physical exertion, as well as in a significant increase in the alpha rhythm in the left hemisphere.

We have shown that freestyle wrestlers are mainly characterized by a hyperthymic type of character accentuation, which is described by activity, vigor, optimism, carelessness, and according to literature data also correlates with the mobility (lability) of the nervous system. Wrestlers with a hyperthymic type of character are characterized by a lower frequency of theta rhythm. After physical exertion, they are characterized by an increase in theta rhythm, whereas the wrestlers with low hyperthymia are characterized by a decrease in this parameter. The situation associated with the wrestling may turn the calm wakefulness state to state of “readiness” in a wrestling situation as well as a possible adaptation of the athlete’s functional system mechanism for the effective implementation of sports activities due to tension of regulatory systems.

Thus, within the framework of the conducted research, we can put forward an assumption about the formation of a specific functional system in professional freestyle wrestlers that performs a regulatory function of adaptation of the organism to the wrestling match. This functional system includes multilevel brain structures, both cortical and subcortical, which are involved in the implementation of various cognitive functions and emotional responses. Calm wakefulness of wrestlers is characterized by the relaxation of this functional system, while the wrestling situation leads to its sharp stressing, which is reflected in the activation of various brain structures, in particular, the anterior cingulate gyrus.

## Figures and Tables

**Table 1:** Electroencephalogram (EEG) rhythm ranges used in the study.

Rhythm	Rhythm Frequency	EEG Electrodes
Theta	4–8 Hz	F3, Fz, and F4
Alpha	8–14 Hz	P3, Pz, and P4
Beta LF	14–20 Hz	C3, Cz, and C4
Beta HF	20–35 Hz	C3, Cz, and C4

**Table 2:** Scores of Well-being, Activity, Mood (WAM) questionnaire in group 1 and group 2 before and after exercises.

WAM Scale	State	Group 1	Group 2	*p*-Value for M-W Criterion	Cohen’s d
Health	Before exercise	5.77	5.77	0.714	0.00
	After exercise	4.75	3.13	0.095	2.31
Activity	Before exercise	5.62	4.9	0.262	0.90
	After exercise	4.8	3.37	0.048 *	2.10
Mood	Before exercise	5.95	5.27	0.381	0.90
	After exercise	5.45	4.33	0.167	1.70

* *p* ≤ 0.05.

**Table 3:** Dynamics of theta rhythm before and after exercise.

Theta Rhythm	Eyes Condition	Before Exercise	After Exercise	*p*-Value for *t*-Test	Cohen’s d
***electrode F3***					
Dominant frequency	BCE	5.42	4.50	0.07	0.64
	BOE	4.83	4.51	0.42	0.47
Average frequency	BCE	5.56	5.18	0.05 *	0.74
	BOE	5.36	5.40	0.84	0.14
***electrode Fz***					
Dominant frequency	BCE	5.29	4.46	0.05 *	0.59
	BOE	4.92	4.44	0.37	0.44
Average frequency	BCE	5.57	5.50	0.72	0.16
	BOE	5.37	5.44	0.65	0.22
***electrode F4***					
Dominant frequency	BCE	5.57	4.14	0.01 **	1.05
	BOE	4.41	4.68	0.58	0.66
Average frequency	BCE	5.63	5.19	0.01 **	1.14
	BOE	5.35	5.30	0.79	0.16

* *p* ≤ 0.05; ** *p* ≤ 0.01.

**Table 4:** Dynamics of theta rhythm before and after exercise in group 1 and group 2 (dominant frequency).

State	Eyes Condition	Group 1	Group 2	*p*-Value for M-W Criterion	Cohen’s d
***electrode F3***					
Before exercise	BCE	4.99	6.26	0.26	1.06
	BOE	4.63	5.23	0.38	0.77
After exercise	BCE	4.05	5.40	0.02 *	3.46
	BOE	4.75	4.02	0.02 *	1.24
***electrode Fz***					
Before exercise	BCE	5.21	5.44	0.91	0.15
	BOE	4.28	6.22	0.02 *	3.09
After exercise	BCE	4.45	4.47	0.38	0.03
	BOE	4.64	4.04	0.05 *	1.16
***electrode F4***					
Before exercise	BCE	5.48	5.74	0.91	0.18
	BOE	4.32	4.6	0.55	0.61
After exercise	BCE	4.15	4.12	0.91	0.17
	BOE	4.99	4.06	0.10	0,.97

* *p* ≤ 0.05.

**Table 5:** Dynamics of alpha rhythm before and after exercise.

Alpha Rhythm	Eyes Condition	Before Exercise	After Exercise	*p*-Value for *t*-Test	Cohen’s d
***electrode P3***					
Dominant frequency	BCE	10.08	10.60	0.05 *	0.94
	BOE	8.86	9.80	0.19	0.80
Average frequency	BCE	10.36	10.69	0.04 *	0.79
	BOE	10.14	10.40	0.15	0.72
***electrode Pz***					
Dominant frequency	BCE	9.94	10.47	0.25	0.56
	BOE	8.66	9.54	0.09	0.86
Average frequency	BCE	10.46	10.69	0.16	0.47
	BOE	10.02	10.37	0.03 *	1.13
***electrode P4***					
Dominant frequency	BCE	10.11	9.81	0.50	0.43
	BOE	9.05	9.75	0.19	0.64
Average frequency	BCE	10.33	10.59	0.19	0.35
	BOE	10.06	10.41	0.08	0.72

* *p* ≤ 0.05.

## Appendix A

**Table A1:** Scores of Shmishek-Leonhard Questionnaire.

Subjects	SCALES										
	A	B	C	D	E	F	G	H	I	J	FALSE
1	24 *	3	9	3	14	10	14	3	9	18 *	4
2	21 *	6	12	9	12	14	16	5	6	24 *	3
3	21 *	12	15	9	12	16	20 *	6	12	24 *	6
4	24 *	6	3	9	12	12	8	6	12	12	2
5	21 *	9	6	12	10	16	6	0	3	12	5
6	21 *	6	6	12	16	10	12	6	6	18 *	2
7	9	6	12	0	10	14	2	3	3	6	2
8	9	15	15	12	4	14	10	3	3	18 *	2
9	9	12	12	9	8	14	8	12	6	12	4

* high values of accentuation scales. Scales: A—Hyperthymia; B—Dysthymia; C—Cyclotimity; D—Emotionality; E—Demonstrativeness; F—Stuck; G—Pedantry; H—Anxiety; I—Excitability; J—Exaltedness.
